# Supplementary material for: Operational Definitions of Polypharmacy and Their Association with All-Cause Hospitalization Risk: A Conceptual Framework Using Administrative Databases
Source: Pharmacy (Basel). 2025 Feb 2;13(1):15. doi: 10.3390/pharmacy13010015 (PMC11859879; doi:10.3390/pharmacy13010015)
Supplement: Supplementary file 1 [file pharmacy-13-00015-s001.zip › pharmacy-3444544 Scotti_et_al_Supplementary.pdf]

pharmacy

MDPI

Article

Operational Definitions of Polypharmacy and Their Association with All-Cause Hospitalization Risk: A Conceptual Framework Using Administrative Databases

Stefano Scotti [1,2], Lorenza Scotti [3], Federica Galimberti [2], Sining Xie [1], Manuela Casula [1,2,*], and Elena Olmastroni [1,2]

[1] Epidemiology and Preventive Pharmacology Service (SEFAP), Department of Pharmacological and Biomolecular Sciences, University of Milan, Milan, Italy
[2] IRCCS MultiMedica, Sesto San Giovanni, Italy;
[3] Department of Translational Medicine, University of Piemonte Orientale UPO, Novara, Italy
* Correspondence: manuela.casula@unimi.it

Abstract: Polypharmacy, defined as the concurrent use of multiple medications, increases the risk of various adverse outcomes. However, the variability in definitions across the literature contributes to substantial heterogeneity. This study aimed to review and characterize different operational definitions of polypharmacy and assess their association with all-cause hospitalization. Data from the pharmacy-refill and hospitalization databases of the Local Health Unit (LHU) of Bergamo, Lombardy, were analyzed. Patients aged ≥40 with at least one reimbursed drug prescription in 2017 were included. Prescription coverage was evaluated using total defined daily doses (DDDs), and all-cause hospitalizations from January to June 2018 were considered. Definitions explored included: (i) WHO's criterion of ≥5 medications by ATC 4th level code; (ii) excluding prescriptions usually for short-term treatments; and (iii) drugs with cumulative annual DDD≥60. Approaches were assessed annually, quarterly, and monthly, and logistic regression was used to estimate odds ratios (OR) for hospitalization risk. Among 431,620 patients, the DDD≥60 definition showed the least variability (20.47%-21.16%) and identified an older, more complex cohort. All definitions showed a dose-dependent association with hospitalization risk. Different definitions of polypharmacy result in varying prevalence, with DDD≥60 being the most consistent. A patient-centric approach is crucial to assess the appropriateness of polypharmacy.

Keywords: Polypharmacy; Drug exposure; Operational definitions; Administrative databases; Epidemiologic methods; Hospitalization risk

Supplementary Material

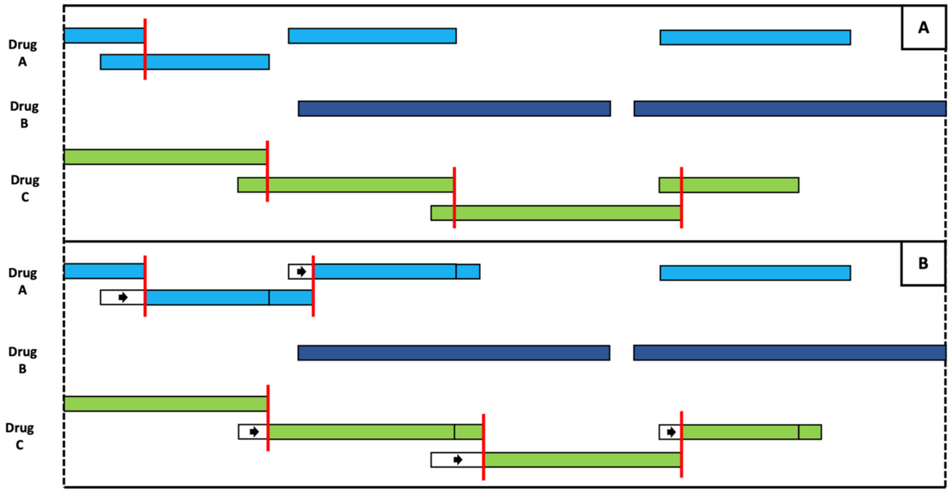

Supplementary Figure 1. Illustrative example of coverage period shifting (Panel B) when drugs were dispensed before the end of the previous coverage period (Panel A), calculated using Defined Daily Doses (DDDs).

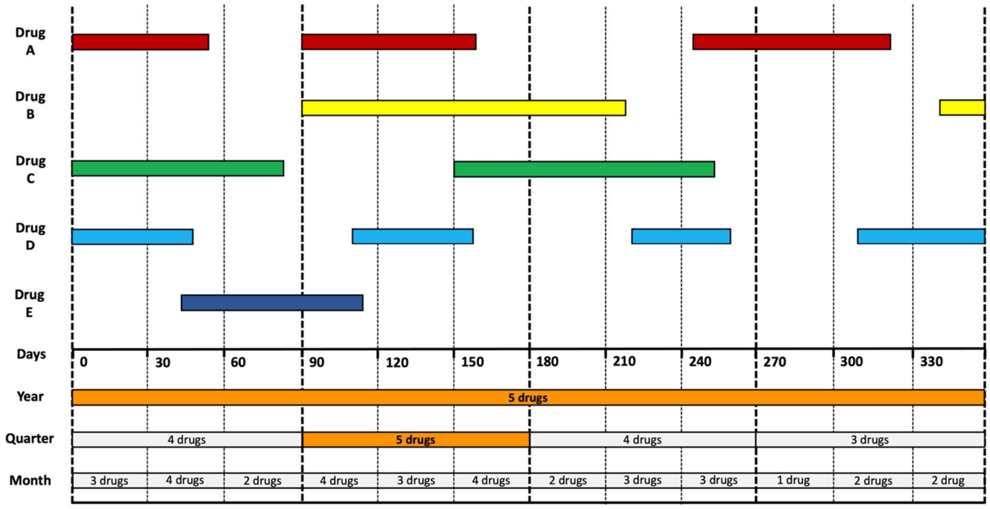

Supplementary Figure 2. Illustrative example of polypharmacy calculation for a patient taking five different drugs (ATC 4th level) within predefined time windows: one year, one quarter, and one month.

Supplementary Table 1. Most frequently prescribed drugs and drug classes in the analyzed cohort. N indicates the number of patients who received at least one prescription for the corresponding drug class during the study period.

ATC 4th level	Drug class	Frequency (N)
A02BC	Proton pump inhibitors	149,574
A11CC	Vitamin D and analogues	110,845
C10AA	HMG-CoA reductase inhibitors	94,384
J01CR	Beta-lactamase resistant penicillins	92,486
J01MA	Fluoroquinolone antibiotics	80,205

C07AB	Beta-blocking agents, cardioselective	77,992
B01AC	Antithrombotic agents, heparins	59,327
C09AA	ACE inhibitors, plain	55,566
C08CA	Calcium channel blockers, dihydropyridine derivatives	53,361
H02AB	Corticosteroids for systemic use, glucocorticoids	52,533
J01FA	Macrolides, lincosamides, and streptogramins	50,426
C09CA	ACE inhibitors, combinations	44,115
C09DA	ACE inhibitors with calcium channel blockers	43,054
M01AE	Non-steroidal anti-inflammatory drugs (NSAIDs), propionic acid derivatives	39,235
N06AB	Antidepressants, selective serotonin reuptake inhibitors (SSRIs)	37,258
R03BA	Beta2 sympathomimetics, short-acting	34,553
J01DD	Cephalosporins, 3rd generation	34,040
B01AB	Antithrombotic agents, oral vitamin K antagonists	31,986
A10BA	Insulins and analogues, fast-acting	31,703
C03CA	Diuretics, potassium-sparing agents	30,981
G04CA	Anticholinergics used in urinary disorders	29,414
R03AK	Leukotriene receptor antagonists	26,593
C09BA	Angiotensin II antagonists, plain	25,771
A07AA	Antidiarrheal agents, excluding antibiotics	25,399
M01AB	Non-steroidal anti-inflammatory drugs (NSAIDs), acetic acid derivatives	24,982
H03AA	Thyroid hormones	24,876
M04AA	Drugs for gout	22,154
J01XX	Antibiotics, other	20,235
M01AH	Non-steroidal anti-inflammatory drugs (NSAIDs), heteroaryl acetic acid derivatives	18,975
A02BX	Drugs for acid-related disorders, combinations excluding antacids	18,466
B03BB	Iron, parenteral preparations	18,082
N06AX	Other antidepressants	17,680
J01CA	Penicillins with extended spectrum	16,950
N02AJ	Non-opioid analgesics, combination products	16,286
A12AX	Other vitamin B preparations	16,120
N03AX	Other antiepileptics	15,902
B03AA	Iron preparations, oral	15,403
N02AX	Other analgesics and antipyretics	14,600
A10BB	Insulins and analogues, long-acting	13,678
B01AA	Antithrombotic agents, platelet aggregation inhibitors	13,441

R03AC	Anticholinergic bronchodilators	12,687
N02AA	Opioid analgesics	12,002
G04CB	5-alpha-reductase inhibitors	11,451
M01AX	Other non-steroidal anti-inflammatory drugs (NSAIDs)	10,975
C01DA	Cardiac glycosides	10,929
M01AC	Non-steroidal anti-inflammatory drugs (NSAIDs), acetic acid derivatives	10,864
C09BB	Angiotensin II antagonists, combinations	10,816
C02CA	Alpha-blockers, combinations	10,528
R06AX	Antihistamines for systemic use	10,481
A10AE	Antidiabetic agents, combinations of oral drugs	10,126
J02AC	Antifungal agents, imidazole derivatives	10,092
A02AD	Antacids	10,081
N05AH	Anxiolytics, sedatives, and hypnotics, combinations	9,847
C10AX	Other lipid-lowering agents	9,829
R03BB	Beta2 sympathomimetics, long-acting	9,698
M05BA	Drugs for the treatment of osteoporosis	9,607
R06AE	Antihistamines, second generation	9,414
S01ED	Antiinfectives for ophthalmic use	9,138
C03DA	Thiazide diuretics	9,030
A10AB	Insulins and analogues, mixed preparations	8,110
J05AB	Antiviral agents for systemic use	7,243
C03EA	Diuretics, potassium-sparing agents, combinations	7,197
A07EC	Antidiarrheal agents, antispasmodics	6,806
B01AF	Antithrombotic agents, direct thrombin inhibitors	6,785
A12BA	Other mineral supplements	6,756
A10BD	Insulins and analogues, biphasic preparations	6,604
C09DB	Angiotensin II antagonists with diuretics	6,581
J01EE	Cephalosporins, 5th generation	6,111
C01BD	Beta-blockers, non-cardioselective	6,065
C07BB	Beta-blockers, cardioselective with diuretics	5,996
A05AA	Bile acids and derivatives	5,886
S01EE	Ophthalmic anti-inflammatory agents	5,723
N06AA	Antidepressants, tricyclic and related antidepressants	5,611
N02CC	Analgesics, opioids	5,400
C01BC	Antiarrhythmics, class I and III	5,322
C10BA	HMG-CoA reductase inhibitors, combinations	5,157

C07AG	Beta-blockers, non-cardioselective with calcium channel blockers	5,144
C10AB	HMG-CoA reductase inhibitors, combinations with other lipid-lowering agents	4,887
B03BA	Iron and folic acid	4,548
D05AX	Other dermatological agents for psoriasis	4,474
R03AL	Antihistamines, combinations with decongestants	4,457
C07AA	Beta-blockers, cardioselective	4,331
C07CB	Beta-blockers with diuretics	3,816
S01EC	Antiinfectives for ophthalmic use, combinations	3,791
P01BA	Anthelmintics	3,689
L04AX	Immunosuppressants, combinations	3,665
L02BG	Antineoplastic agents, alkylating agents	3,580
B01AE	Antithrombotic agents, oral vitamin K antagonists	3,557
C08DB	Calcium channel blockers, combinations with diuretics	3,466
A02BA	Antacids, aluminium compounds	3,423
N04BA	Antiparkinson drugs, levodopa and combinations	3,400
A12AA	Vitamin B1 and B12 preparations	3,321
C01EB	Antiarrhythmics, class III	3,265
B02AA	Antithrombotic agents, antiplatelet agents	3,259
M05BB	Drugs for the treatment of osteoporosis, selective estrogen receptor modulators	3,248
N02AB	Opioid analgesics, combinations	3,189
G03CA	Estrogens, natural and synthetic	3,100
C01AA	Antiarrhythmics, class I	3,000
C08DA	Calcium channel blockers, combinations with beta-blockers	2,996
R03DC	Combination products for respiratory diseases	2,987
N03AG	Antiepileptics, combinations	2,892
N03AF	Antiepileptics, other	2,616
J01DB	Cephalosporins, 2nd generation	2,586
A10BH	Antidiabetic agents, combinations with other antidiabetic agents	2,519
C09BX	Angiotensin II antagonists with diuretics	2,503
N04BC	Antiparkinson drugs, non-dopamine receptor agonists	2,493
N06DA	Antidepressants, serotonin-norepinephrine reuptake inhibitors (SNRIs)	2,467
A10BX	Antidiabetic agents, combinations with insulin	2,411
B05BB	Blood substitutes and plasma protein fractions	2,175
C02AC	Alpha-blockers, plain	2,143
H03BB	Thyroid hormones, combinations	2,128

C03BA	Diuretics, thiazide-like	1,979
N05AX	Anxiolytics, sedatives, and hypnotics, other	1,975
N01BB	Anesthetics, general	1,966
J01DC	Cephalosporins, 4th generation	1,954
C03AA	Diuretics, thiazides	1,904
A03FA	Antispasmodic and anticholinergic drugs	1,891
A07EA	Antidiarrheal agents, adsorbents	1,842
C03EB	Diuretics, other combinations	1,832
L02AE	Antineoplastic agents, mitotic inhibitors	1,806
A04AA	Antiemetics and antinauseants	1,793
N05AD	Antipsychotics, atypical	1,717
J01AA	Penicillins, narrow spectrum	1,708
N06DX	Other antidepressants, miscellaneous	1,621
N03AE	Antiepileptics, carbamazepine	1,608
N05AL	Antipsychotics, typical	1,485
A10BJ	Insulins and analogues, ultra-fast acting	1,306
B03XA	Iron and other agents for anemia	1,305
P01BC	Anthelmintics, combinations	1,266
A06AD	Laxatives, osmotic	1,250
A09AA	Digestive enzymes	1,237
A10BG	Insulins and analogues, premixed	1,198
N03AA	Antiepileptics, valproic acid and derivatives	1,179
N05AN	Antipsychotics, atypical	1,169
L02BA	Antineoplastic agents, alkylating agents	1,168
L02BB	Antineoplastic agents, other	1,115
L04AD	Immunosuppressants, calcineurin inhibitors	1,106
M05BX	Other drugs for osteoporosis	1,086
L03AA	Immunostimulants	1,084
N04BD	Antiparkinson drugs, COMT inhibitors	1,084
D01BA	Antifungal agents, imidazole derivatives	1,067
G03CX	Progestogens and estrogens in combination	1,066
A10BK	Insulins and analogues, combinations with GLP-1 receptor agonists	1,053
M04AC	Drugs for the treatment of gout, uricosurics	1,026
P02CA	Anthelmintics, benzimidazoles	1,015
A03BB	Antispasmodics, combinations	1,007
B03AB	Iron preparations, oral	928
G02CB	Uterine relaxants, combinations	898
B05BA	Blood substitutes and plasma protein fractions	890
V03AE	Contrast media	862
S01EA	Antiinfectives for ophthalmic use, combinations	845
G03DA	Progestogens	837

N04AA	Antiparkinson drugs, dopaminergic agents	810
G03DB	Progestogens, synthetic	807
D11AX	Dermatological agents for acne	803
A10BF	Insulins and analogues, ultra-long-acting	793
A10AD	Insulins and analogues, rapid-acting	792
R03DA	Inhaled corticosteroids, combinations	773
L01XX	Other antineoplastic agents	702
D07AC	Corticosteroids for dermatological use	686
P01AB	Anthelmintics, other	671
D07AD	Corticosteroids for dermatological use, combinations	607
N05AA	Antipsychotics, typical (1st generation)	602
M03BX	Antispasmodics for gastrointestinal use	596
L01BA	Antineoplastic agents, alkylating agents	574
S01XA	Ophthalmic anti-inflammatory agents, other	548
G04BD	Alpha-blockers, urologic	524
R05DA	Expectorants, combinations	519
N02AE	Non-opioid analgesics, combination products	510
B01AX	Antithrombotic agents, combinations	505
G03FA	Androgens and anabolic steroids	496
J06BB	Immunostimulants, vaccines	440
H05BX	Parathyroid hormone and analogues	381
G03DC	Progestogens, combinations	375
G04BE	Drugs for erectile dysfunction	368
J01FF	Other antimicrobial agents	336
C09XA	Angiotensin II antagonists, combinations with other antihypertensive agents	333
D06BX	Antifungals for dermatological use	296
G03GA	Progestogens, natural	294
L04AA	Immunosuppressants, calcineurin inhibitors	286
G03XB	Progestogens, other	284
H05AA	Parathyroid hormone and analogues, recombinant	278
A02BB	Antacids, calcium carbonate	275
A02BD	Antacids, magnesium hydroxide	274
G03FB	Progestogens, combinations with estrogens	273
N07BB	Drugs used in addictive disorders	271
J04AB	Antifungal agents, terbinafine	251
C10AC	HMG-CoA reductase inhibitors, combinations	244
N07AA	Drugs used in hyperactivity and attention disorders	235
B02BA	Antithrombotic agents, anticoagulants	232

G03HB	Estrogens, conjugated	213
H01CB	Growth hormone and analogues	210
C09DX	Angiotensin II antagonists, combinations with calcium channel blockers	205
J01GB	Penicillins with beta-lactamase inhibitors	203
D05BB	Other dermatological agents for acne	199
D07AB	Topical corticosteroids, combinations with antimicrobials	190
S01AD	Ophthalmic anti-inflammatory agents, corticosteroids	179
B05AA	Blood substitutes and plasma protein fractions, albumin	161
J05AF	Antiviral agents, nucleoside and nucleotide analogues	158
L02AB	Antineoplastic agents, alkylating agents	154
L01AA	Antineoplastic agents, cytotoxic	150
P02CC	Anthelmintics, other	133
R03BC	Long-acting beta2 agonists	133
N03AB	Antiepileptics, barbiturates	131
J01MB	Cephalosporins, combinations with other antimicrobial agents	130
H01BA	Thyroid hormones, thyroid gland extracts	123
D06BB	Dermatological agents for eczema	112
R05DB	Cough preparations, combinations with expectorants	112
H04AA	Corticosteroids for ophthalmic use	106
L02BX	Antineoplastic agents, other	86
G03XC	Progestogens, combinations with estrogens	83
N07XX	Drugs used in other neuropsychiatric disorders	78
G03BA	Estrogens, estradiol	73
J01DE	Cephalosporins, 4th generation	64
J04AC	Antifungal agents, imidazole derivatives	64
N05AB	Antipsychotics, phenothiazines	63
G03HA	Estrogens, natural	61
A10AC	Antidiabetic agents, combinations with other antidiabetic agents	59
C02AB	Alpha-blockers, selective	57
N04BX	Antiparkinson drugs, other dopamine receptor agonists	51
N02CX	Non-opioid analgesics, combination products	49
J01CE	Cephalosporins, 3rd generation	48
H01AC	Thyroid hormones, other	43
G03GB	Estrogens, other	39

D10BA	Retinoids	38
J01XA	Other penicillins	37
J04AK	Antifungal agents, polyenes	36
J04AM	Antifungal agents, azoles	33
A03AX	Antispasmodics, combinations with other agents	33
P02DA	Anthelmintics, other	32
N05AG	Antipsychotics, atypical (2nd generation)	32
G03XA	Progestogens, non-synthetic	27
L03AB	Immunostimulants, interleukins	26
N05AF	Antipsychotics, atypical (newer)	24
A16AA	Appetite suppressants	23
N05AC	Antipsychotics, traditional	21
C01BA	Antiarrhythmics, class I	20
A01AB	Antiseptic and disinfectant solutions	18
N02BA	Opioid analgesics	16
L01CB	Antineoplastic agents, alkylating agents	16
V03AC	Contrast media	15
A06AH	Laxatives, stimulants	15
N06BA	Antidepressants, serotonin-norepinephrine reuptake inhibitors (SNRIs)	14
R03DX	Combination respiratory agents	14
S01EB	Ophthalmic anti-inflammatory agents, corticosteroids	14
C01CA	Antiarrhythmics, class I	13
L01BB	Antineoplastic agents, platinum derivatives	12
N05AE	Antipsychotics, atypical (2nd generation)	12
J01CF	Macrolides and lincosamides	8
N02BE	Opioid analgesics, combinations with non-opioid analgesics	7
J01XD	Other penicillins	7
M01CB	Non-steroidal anti-inflammatory drugs (NSAIDs), cyclooxygenase-2 inhibitors	6
N03AD	Antiepileptics, carbamazepine derivatives	4
V03AH	Contrast media, other	4
G02AB	Uterine relaxants	3
G03AA	Estrogens, conjugated	2
L01XB	Antineoplastic agents, targeted therapies	2
N02AD	Non-opioid analgesics	2
N07BC	Drugs for attention deficit hyperactivity disorder	2
R05CB	Cough preparations, antitussives	2
V04CD	Diagnostic agents, radiopharmaceuticals	2
B02BD	Antithrombotic agents, thrombolytics	2

J02AB	Antifungal agents, triazoles	1
N02CA	Non-opioid analgesics, other	1
N05CD	Anxiolytics and sedatives	1
R06AD	Antihistamines, combinations with other agents	1

Supplementary Table 2. Prevalence of patients in polypharmacy defined as the use of five or more medications without considering the overlap of drugs within the same therapeutic class nor considering the coverage of the prescription (classical method, see methods section).

	**All ATC**	**Chronic ATC**	**DDD≥60**
Annual (%)	40.05	33.54	21.16
Quarter (%)	32.51	28.46	20.04
Month (%)	26.26	23.78	18.15

Supplementary Table 3. Prevalence of patients in polypharmacy (5 or more drugs). Monthly and quarterly measures of polypharmacy over the year is reported as the average number of dispensed drugs between months and quarters.

	**All ATC**	**Chronic ATC**	**DDD≥60**
Annual (%)	39.98	33.51	21.16
Quarter (%)	26.59	24.39	19.43
Month (%)	20.27	19.35	17.08

Supplementary Table 4. Prevalence of patients in polypharmacy defined on monthly base as having at least 50%, 80%, or 100% of the follow-up period (1 year, 2017) covered by five or more medications.

	**All ATC**	**Chronic ATC**	**DDD≥60**
50% (6 out of 12 months) (%)	17.78	17.18	15.77
80% (10 out of 12 months) Annual (%)	12.28	11.94	11.16
100% (12 out of 12 months) Annual (%)	8.15	7.94	7.48

Supplementary Table 5. Adjusted estimates (odds ratios (OR) and 95% confidence intervals [LCL: lower confidence limit, UCL: upper confidence limit]) for the association between polypharmacy classes and the risk of hospitalization for all-causes considering only hospitalizations longer than 7 days.

**Polypharmacy classes**	**Maximum number of dispensed drugs**		
	**Annual**	**Quarter**	**Month**

		N	%	N	%	N	%
All ATC	<5	259,047	60.02	316,845	73.41	344,126	79.73
	5	37,530	8.70	31,737	7.35	28,353	6.57
	6	30,396	7.04	24,372	5.65	20,760	4.81
	7	24,443	5.66	18,145	4.20	14,485	3.36
	8	19,424	4.50	13,524	3.13	9,637	2.23
	9	15,235	3.53	9,428	2.18	6,116	1.42
	≥10	45,545	10.55	17,569	4.07	8,143	1.89
Chronic ATC	<5	287,005	66.49	326,329	75.61	348,085	80.65
	5	34,241	7.93	30,003	6.95	27,405	6.35
	6	27,015	6.26	22,791	5.28	20,157	4.67
	7	21,467	4.97	17,027	3.94	13,941	3.23
	8	16,568	3.84	12,327	2.86	9,081	2.10
	9	12,721	2.95	8,525	1.98	5,629	1.30
	≥10	32,603	7.55	14,618	3.39	7,322	1.70
DDD≥60	<5	340,283	78.84	347,760	80.57	357,886	82.92
	5	26,752	6.20	26,003	6.02	25,211	5.84
	6	20,116	4.66	19,218	4.45	18,118	4.20
	7	14,691	3.40	13,837	3.21	12,165	2.82
	8	10,289	2.38	9,312	2.16	7,867	1.82
	9	7,066	1.64	6,204	1.44	4,678	1.08
	≥10	12,423	2.88	9,286	2.15	5,695	1.32

Disclaimer/Publisher's Note: The statements, opinions and data contained in all publications are solely those of the individual author(s) and contributor(s) and not of MDPI and/or the editor(s). MDPI and/or the editor(s) disclaim responsibility for any injury to people or property resulting from any ideas, methods, instructions or products referred to in the content.
